# ‘What do we *do*, doctor?’ Transitions of identity and responsibility: a narrative analysis

**DOI:** 10.1007/s10459-020-09959-w

**Published:** 2020-01-20

**Authors:** Sarah Yardley, Ruth Kinston, Janet Lefroy, Simon Gay, Robert K. McKinley

**Affiliations:** 1 Keele University School of Medicine, Keele, UK; 2 Palliative Care Service, Central and North West London NHS Foundation Trust, St Pancras Hospital, 5th Floor South Wing, 4 St. Pancras Way, London, NW1 0PE UK; 3 University of Leicester School of Medicine, Leicester, UK

**Keywords:** Education, Medical, Qualitative research, Professional practice, Transition, Identity, Responsibility, Narrative analysis

## Abstract

Transitioning from student to doctor is notoriously challenging. Newly qualified doctors feel required to make decisions before owning their new identity. It is essential to understand how responsibility relates to identity formation to improve transitions for doctors and patients. This multiphase ethnographic study explores realities of transition through anticipatory, lived and reflective stages. We utilised Labov’s narrative framework (Labov in J Narrat Life Hist 7(1–4):395–415, 1997) to conduct in-depth analysis of complex relationships between changes in responsibility and development of professional identity. Our objective was to understand how these concepts interact. Newly qualified doctors acclimatise to their role requirements through participatory experience, perceived as a series of challenges, told as stories of adventure or quest. Rules of interaction within clinical teams were complex, context dependent and rarely explicit. Students, newly qualified and supervising doctors felt tensions around whether responsibility should be grasped or conferred. Perceived clinical necessity was a common determinant of responsibility rather than planned learning. Identity formation was chronologically mismatched to accepting responsibility. We provide a rich illumination of the complex relationship between responsibility and identity pre, during, and post-transition to qualified doctor: the two are inherently intertwined, each generating the other through successful actions in practice. This suggests successful transition requires a supported period of identity reconciliation during which responsibility may feel burdensome. During this, there is a fine line between too much and too little responsibility: seemingly innocuous assumptions can have a significant impact. More effort is needed to facilitate behaviours that delegate authority to the transitioning learner whilst maintaining true oversight.

## Introduction

In order to learn optimally medical students need to understand the complexities of clinical workplaces and how best to engage in workplace activities (de Feijter et al. 2011; Mennin 2010). There is a fine balance to be found between not enough and too much autonomy in this process (Cruess et al. 2016). This is particularly so at points of transition (Cruess et al. 2014; Yardley et al. 2018).

This is the second paper from a multiphase ethnographic study in a UK locality. Our study sought to both describe the realities of transition from undergraduate medicine into clinical practice and answer questions about the relationship between identity formation and changes in responsibility. We previously published a realist evaluation of the transition to qualified doctor, moving from anticipation, through lived experience, to post hoc reflection (Lefroy et al. 2017). This confirmed that identity and accepting responsibility are intertwined and critically significant to readiness for practice. In brief, we demonstrated that:

- Transition experiences of ‘firsts’ (the first time something happened or was required of a newly qualified doctor), create a step-change in perceptions of responsibilities associated with student and doctor identities (Lefroy et al. 2017).
- Transition is experienced as a process despite being felt most acutely at the point where the newly qualified doctor first makes what they believe are largely autonomous decisions. They report feeling that they are required to make decisions before they fully own their new identity (Lefroy et al. 2017).
- Responsibility is intertwined with identity formation and this process is important in its own right (Lefroy et al. 2017).

Our realist evaluation produced a mid-range theory which proposes that new doctors are able to face and hence learn from ‘firsts’ in practice when, among other things, their internal and external sense of responsibility cohere. Mid-range theories are concepts that explain specific context, mechanisms and outcomes configurations (CMOCs) within the overall programme theory (Wong et al. 2012). In ideal circumstances, giving and acceptance of increasing responsibility should be a gradual process, combining appropriate support with appropriate challenges that enable new doctors to confirm their abilities. Without both support and challenge unsafe practice could result, with newly qualified doctors at high risk of developing avoidant and/or dangerous practices when faced with firsts (Lefroy et al. 2017).

The giving of responsibility during student workplace experience is an important mechanism for building self-efficacy prior to the changes in expectation and authority (e.g. prescribing) associated with being a qualified doctor (Illing et al. 2013). However, our realist evaluation demonstrated that total prior preparedness for responsibility is not a realistic goal. The new doctor’s sense of identity prevents this: identifying oneself as the frontline clinician, introducing oneself as a doctor, signing prescriptions or other items requiring the authority of a doctor and responding when someone wanted to speak to the doctor were consistently identified as significant post-qualification firsts. This supports the need for a clear role with real responsibility in a team to support transfer of learning from being an undergraduate into working as a doctor, a role called ‘the assistantship’ in the UK. There is, however, no set model for how medical schools and clinical workplaces deliver such assistantships, and the GMC does not define what specific duties, level of supervision or responsibilities should be included (Crossley and Vivekananda-Schmidt 2015; GMC 2011). Given that identity and responsibility are mediated through contextually situated social processes and can be declined, claimed and/or withheld, or conferred (Illing and Crampton 2015; Dornan et al. 2007) this warranted further analysis.

In this paper we provide an in-depth analysis of these issues unpacking the facets of responsibility and identity found in our large data set of audio diaries, interviews and focus groups with people as they made the transition from medical student to doctor. We drew on two additional narrative techniques for this new and more detailed analysis:

- Data, thematically coded by multiple researchers, was extracted from the codes/subcodes of ‘issues of responsibility’ and ‘issues of identity’ using a matrix in NVivo Version 10.0 (QSR International Pty Ltd, Melbourne, Vic, Australia) to identify a cross-matched data subset.
- We used a storytelling analytic tool, the Labov framework (Labov 1997) to provide a new lens through which to view the data with focus on meaning constructed by participants when telling stories about their experience. This framework isolates recurring narrative structures that help us understand how people encode information about the world on a personal level. This facilitates thematic analysis of form and content in order to recognise recurring meanings or themes. We sought to understand the meaning of that meaning, undertake a detailed look at self-presentation of the speaker, identify potential mismatch between speakers’, listeners’ and supervisors’ expectations, and to identify what the storyteller wished to convey in telling a story in a particular way. We wanted to understand how the different elements (e.g. personal ethics and values, professional duties, internal and external expectations) found in the conceptualisation of responsibility and of identity interact.

## Objectives

To understand:

1. How those transitioning make sense of two intertwined concepts: responsibility and identity.
2. The intersection between responsibility and identity during transition with resultant impact on safe medical practice.

## Methods

### Theoretical orientation

We drew on both objective idealism (i.e. there is a world of collectively shared understandings) and critical realism (i.e. knowledge of reality is mediated by perceptions and beliefs) in this narrative analysis (Barnett-Page and Thomas 2009). These perspectives are complementary and closely related (Spencer et al. 2003).

### Study design

We generated data from final year medical student participants (n = 32), 11 of whom were followed through transition to the role of doctor and from 70 trainee doctors (within 3 years post-graduation) from 17 different UK medical schools. Data was generated using logbooks and audio-diaries, followed by audio-recorded participant interviews exploring their diary entries and/or focus groups. Anonymised verbatim transcriptions of audio were prepared for thematic analysis (Lefroy et al. 2017).

### Data generation

We used NVivo to search the original data set (32 medical students, 70 postgraduate trainee doctors) for all data with a responsibility code, sub-theme or theme cross-matched with all data coded to an identity code, subtheme or theme. The number of data extracts by cross-matched theme is shown in Table 1.

**Table 1:** Cross-matched coding matrix of responsibility and identity codes, sub-themes and themes

	Identity-global theme	Identity-conferred by others	Becoming the doctor	Descriptions of identity
Responsibility—global theme	2	6	6	5
Responsibility for clinical decision-making	0	18	28	8
In your name as the doctor	1	18	13	10
Taking responsibility (stepping forward) versus being given it or not having it	1	29	38	21
Total data extracts	204			

Numbers indicate how many data extracts were coded at each cross point

### Data analysis

Two authors (SY and RK) analysed data extracts with respect to the intersection of responsibility and identity using constant comparison techniques, a data analytic process whereby each finding and interpretation is compared to previous findings as it emerges from the data. Labov’s stages of a story (Labov 1997) was used as a framework to conduct a narrative analysis of participants’ stories about step-changes in responsibility and its interrelationship with their identities (Frank 2010).

Stories may be told in different genres (e.g. adventure, quest, confession, tragedy, victory, defeat or suspense) but regardless of genre, narrative analysis using Labov’s framework allowed us to critically consider what the meaning and purpose of the story might be to the storyteller as well as analysing its content. Labov described six stages of a ‘whole story’, although one or more may be missing in any specific instance (Table 2).

**Table 2:** Labov’s six stages of a ‘whole story’

Stage		Description
1	The abstract	The storyteller gives a cue that the story is about to be told
2	Orientation	Information about time, place, person, characters—from which we can learn what the storyteller considers to be important contextual information to understand what they are going to say
3	A complicating event	Something which happened (such as a ‘first’ for a new doctor) and which demanded a response
4	Resolution	The consequences of the event, for example- successful completion of a first
5	Evaluation	The storyteller’s attitude to events including sense of morality, for example—confidence derived from rising to the challenge but comment on perceived ‘rightness’ of lack of support, such as when working ‘alone’ at night
6	Coda	Moving on from the story to other things
*Stage groupings*		
1–3		Referential elements: orientate and ground the story in its contextual world by referencing events in sequential order as they occurred
3–6		Evaluative elements: describe the storyteller’s purpose in telling the story

Our analysis focused on elements 2–5 paying specific attention to metaphors and language to understand the impact of responsibility on trainees as they experienced it at each transition. We sought to understand the significance of the stories’ meanings for healthcare practice as well as participant learning (Webster and Mertova 2007).

## Results

Results are presented in two stages. First, we discuss the overarching genre used to describe experiences of responsibility. Second, we explore themes identified in relation to the process of transition: anticipatory, the lived experience and post hoc reflection. The excerpts are drawn from all data sources and are tagged to identify their source (Table 3).

**Table 3:** Key to data tags

Respondent	Type of data and individual identifier
Student (in the UK pre-qualification these are undergraduate medical students)	Interview Individual identification numbers are given as student IDxx e.g. student ID29
Foundation year (FY) (in the UK, these are newly qualified doctors, FY1—first year post qualification, FY2—second year post qualification. Intern would be the international equivalent)	Interview Individual identification numbers are given as FYIDxx
Foundation year (FY)	Focus groups Identified by Site (1–3) and Group e.g. 2 Individual identification numbers are given as FGIDxx
Core trainees (CT) (in the UK Core Trainees are equivalent to junior resident)	Focus groups Identified by site (4) and group Individual identification numbers are given as FGCTxx

The excerpts provide snapshots of moments in which participants engaged with the concept of responsibility. While these stories are often brief and fragmented (i.e. frequently do not contain a traditional ‘beginning, middle and end’), they do feature some or all of the narrative elements described by Labov. Excerpts have their ‘story stage’ identified in bracketed text.

## The overarching genre: metastories of adventures and quests

When describing personal experiences of responsibility, our participants’ stories best fit the genres of adventure and/or quest. Challenging situations that must be overcome are described, frequently with a sense of jeopardy for storyteller or patient. Depending on their perception of the responsibility they hold and usually in reference to their stage within the process of transition, the participant may see themselves as the main protagonist, ‘the hero’, or in a more supporting role.

The following excerpt illustrates the genre through a new doctor’s first experience of responsibility for acute care. The referential narrative elements, the abstract, orientation and complication, describe an acute situation where actions were urgently required. The evaluative narrative, the resolution and evaluation that follows, suggests that while the junior doctor takes the predominant role, he does not see himself as acting completely autonomously and responsibility is shared with the senior doctor who reviews and judges his decisions. He is clear that it is the absence of someone more experienced that compels his action. Though he felt trepidation in undertaking the action, he was able to draw on earlier experience as a student. Previously rehearsed ABCDE training drills offered a sense of structure and perhaps reassurance. Success was measured through having achieved all the actions a more senior clinician would have done. With this came a growing sense of capability:

…on AMU [Acute Medical Unit] where I was left to deal with that one myself [abstract]. A patient had been brought up from A&E [Accident and Emergency] with an upper GI [gastrointestinal] bleed, the nurse had wafted the notes in front of my face saying that the early warning score [a numerical indicator of severity] of potential clinical [sic] was over 10, can I come and see him now, and I thought well there’s only four of us on, they’re all busy, I haven’t seen them in a while, I’ll have to go and see him myself [orientation]. Went in and he promptly vomited up 2 litres of blood in front of me, and so I thought I’m going to have to do something quick [complication], I got someone to bleep the registrar [UK equivalent of resident] and then I went into A, B, C, D, E mode,… assessing him thoroughly, knew that blood would take a while, that we probably couldn’t activate major haemorrhage pathway yet, knew that his blood pressure needed maintaining, knew that his haemoglobin needed boosting up, so I ordered the blood and I also ordered albumin as well, which was something that I’d seen done in A&E on my fifth year rotation and I just recalled back to that and thought I’m going to have to do it, there’s nothing else I can do. Started giving him a lot of fluid whilst we were waiting for that ...albumin and blood [resolution]. The registrar rang back after all this had happened, asked me what had happened, what I’d done and he said ‘I can’t do anything else, I’ll come up shortly.’ And he came up, didn’t really change what I’d done, rang the blood bank to speed things up a little bit more and… the patient was okay. He went to endoscopy next morning, was banded and discharge home about a week later [evaluation]. FYID3.

For newly qualified doctors there can be a stark contrast in their experiences as a student. For some the realities of the first weeks of work are harsh, feeling alone in their quests:

…in my first…or in my second week [abstract], but it was only like day three, my senior was off sick for the whole week and there was no consultant [UK equivalent of attending] in the hospital, so it was just me [orientation]. So checking blood results, I’d get obsessed with minute abnormalities [complication] that now I’d just be like ‘well, that’s fine’, but at the time I was like what do I do with this phosphate level –it’d be basically normal, but I wouldn’t know that it was…, I didn’t know what it did, I didn’t know whether I should be worried or not, so you know, stress was…[evaluation] SITE 4, Group 1, FGCT2.

and:

[Interviewer: You make it sound as if you were very alone?]

…with those surgical twilight shifts, it certainly felt like that. I mean you can be quite alone at times [complication], if your SHOs [senior house officer, also known as core trainees, UK equivalent of junior resident] are off, your registrar’s in clinic and things are going wrong and you can’t reach anybody –yeah, definitely, times when you feel very alone [evaluation]. SITE 1, Group 2, FGID3.

These lonely and stressful experiences reach a resolution in our newly qualified doctors’ minds when they concluded that this was the only way to learn and so their quests became rites of passage:

there are a lot of things that you will only learn in those moments, in those horrible, terrifying, heart-wrenching moments when you are an F1…moments when you just, like, you want to cry, but you will not learn those things until it is your time and you’re an F1 and you’re in that horrible situation –that’s the only time you’re going to learn it. …you can prepare a medical student as much as you want, but those moments aren’t going to come until their name is the name that’s going to be signed and they’re the ones that the nurses are looking to for an answer –it’s just not going to happen [resolution]. SITE 1, Group 2, FGID2.

## Stories of transition

Participants’ stories provide both referential and evaluative elements through which they tell of their experiences and their immediate and subsequent impact. We now consider these in terms of the process of transition whereby the student, and those supporting them, describe how they evolve from supported participation in clinical duties to actively adopting responsibility for decision making and task completion.

We then consider the impact of responsibility on the individual and the perceived changes to their internal context and the way they are perceived by their teams.

### Anticipating and preparing for responsibility: understanding roles, requirements and becoming part of the team

Students repeatedly described patient care in terms of a series of challenges to be completed. Success could be recognised externally or internally through a range of outcomes. These include achieving clinically appropriate patient care, being rewarded by gaining access to further learning opportunities, feeling increasingly useful and a growing sense of acceptance and membership of the clinical team. However, even with acceptance and team membership the student’s role remains one of limited responsibility, that of the apprentice or ‘sidekick’. Three years on, doctors retrospectively recall this sense of looking in at responsibility from the outside.

yeah,…obviously there’s an element of practice [orientation] and you can go on on-calls and follow the doctor around and watch them take the bleeps and make up management plans, [complication] but it’s never your…you know, it’s not you…[evaluation] SITE 4, Group 2, FGCT2.

Acquiring an understanding of the future role with its inherent demands and responsibilities is important if students are to engage in appropriate activities and level of challenge. While students perceive the foundation doctor’s role as low in the healthcare hierarchy, they recognise its pivotal role of caregiver and sentry—surveying and reporting important information to more senior members of the clinical team:

So because F1s are…well, I’ll have to know about what… patients have come in [orientation]…because I’m the one who’s going to be, I think, the first point of contact for those patients,…I’ll have to know about why the patient is there, what’s been happening with the patient,…and being able to tell my seniors what’s going on [complication]. Also, another role is just prescribing, that’s a common thing that you have to do on the wards [complication]. And discharge summaries, yeah, that’s a big thing [complication]. Yeah. So the main responsibility is just patient care and making sure the patient is alright and in a safe environment [evaluation]. Student ID29.

Workplace learning occurs through a spectrum of participation. Students seek to observe and then engage in supported practice, but their role and place within the clinical team is context dependent and affected by many factors. These include the stage of learning and required curricular outcomes, who the student perceives as responsible for their supervision, assessment of their performance or judging their clinical competence and the team dynamic including who is immediately available for the care of the patient should that be required. This is rarely made explicit and students may only discover the level of engagement permitted by trying it:

…oh, yeah, I remember trying to ring [abstract], –when I was in A&E –I remember trying to ring the heart failure nurse, ‘cause we had a patient who’d just come in with a past medical history of heart failure and stuff and they were unwell [orientation] at the time, suddenly short of breath and stuff, and they needed to be seen by the heart failure team [complication]. I was actually just…well, somebody next to me, to make sure I was saying the right thing, but I made the call and yeah, they came down, because I said the right thing [resolution]. Student ID06.

The point at which the student is supported to move from observation into more participative learning is not clearly defined. Concerns regarding perceived risk and patient’s welfare and safety are paramount. Trust is a key factor in deciding what activities a student may be allowed to undertake. When a mismatch in expectations occurs, opportunities may be denied outright rather than resolved through provision of appropriate support:

It’s interesting that you talk about the 5th year medical students, although it’s supposed to be an apprenticeship [orientation] as such, at the moment…you tell someone you’re a final year medical student [complication] they go ‘oh okay, I hear medical student, I can’t trust you with anything’ so…for example, ordering investigations –they’ll just go ‘you’re a medical student, you’re not entitled to do this’ [evaluation] SITE 1, Group2, FGID1.

Sometimes students did not step-up to the level of active participation and responsibility expected by their team:

it’s not necessarily more time, it’s using the time productively [orientation] and not just sitting on the side-lines and watching the juniors [complication] but actually being on the ward round and writing in the notes [resolution], taking a little bit more responsibility [evaluation] SITE 4, Group 2, FGCT3.

However, FGID4 offers a clear exposition of identity precluding taking responsibility, believing it can only be conferred by proxy:

I think…yeah, the issue about responsibility –it’s not a case of not wanting to take responsibility, it’s rather not being able to take responsibility. So as [M1] has said, we can go for it and I let my medical students do quite a lot of things [orientation], but when the consultant calls you and says can you justify why you requested this x-ray [complication], that’s my job, it’s not the medical student’s job, so they can do it and go home and they can write whatever they want in the notes and go home, but at the end of the day it’s my decision, you know, to cover that. So as a medical student you don’t have that and you can’t be prepared for that, you have to be on the job [evaluation]. SITE 1, Group 2, FGID4.

Once qualified the requirement to accept responsibility for clinical care becomes inevitable.

Students rarely make decisions affecting patients, their focus being predominantly on developing clinical proficiency and meeting the curriculum’s expectations of them which may prevent full participation:

But they haven’t got time to come to the ward because by the time they’ve gone to this many clinics and this many sessions and done this many tiny little bits and bobs…when can they go? [complication] By that point, the ward round’s ended, we’ve done all our jobs and we’re doing something else and there’s not actually any practical learning that they can then come along to [evaluation] SITE 4, Group 2, FGCT2.

Use of terms such as ‘tiny little bits and bobs’ suggests a perception students’ have misdirected their efforts and are unable to recognise activities which are integral to being a useful team member and developing their capabilities for transition to qualified doctors. The student is seen as an opportunistic learner rather than an active contributor to care while the student perspective is that pressure to meet curricular requirements can subvert their focus on activities which would help them prepare for real clinical practice:

but they’re coming into get the ticks in the book that gets them through [orientation]. SITE1, Group 2, FGID3.

they tend to be quite set in what they want to get out of it [evaluation]. They’ll have a list of things and procedures that they need to get signed off, so they’ll come onto the ward [orientation], get the bloods signed off and then they’ll go [complication]. But that’s not preparing them for what they need to do as an F1, which is the ward rounds and actually seeing patients [evaluation]. SITE 4, Group 2 FGCT2.

Clarity on the role and activities a student should undertake within each setting also appears to be learnt through trial and error as the capabilities and expectations of each student and team are explored. Those supervising are selective about tasks they feel are safe for students to undertake and the degree of autonomy they allow students:

I think…yeah, the issue about responsibility –it’s not a case of not wanting to take responsibility, it’s rather not being able to take responsibility. SITE 1, Group 2, FGID3.

Words like ‘allow’ and ‘stamping it’ suggest those in supervisory roles see themselves as gatekeepers and validators of learning:

FGID1– I remember as an F1 now, I allowed medical students to write the forms and get involved in all the processes…[orientation].

FGID3– yeah, and then just sign it.

FGID1– yeah, so in a sense they are doing it as students….

FGID1– ‘cause all I’m doing is stamping it, saying that I think it’s okay, so I do think…[evaluation].

FGID3– that’s things with forms and that’s all fine, but just seeing a patient by yourself –again you still allow the med student to see the patient and let them report back to you but I would always go back [complication] with them again and pretty much do the examination myself just to double-check [resolution]. Site 1, Group 2.

The requirement to ‘report back’ and the need to ‘double check’ convey hierarchy, desire to safety net and that the doctors perceive they retain responsibility for the activities of those they supervise.

There may be senior clinicians who act as champions, advocating for students and supporting their engagement and integration into the team. This appears to be done by making expectations regarding the level of engagement and supported practice explicit to both student and the wider clinical team:

I think it very much depends on the ward. Because working in infectious diseases, doctor [name] seems very involved [orientation] and she’ll have the students going to take histories, going to take bloods, going for procedures with patients [complication] –I think it depends on the setting and the consultants and the other doctors involved in delivering the education in that setting, how much they get out of it [evaluation]. SITE 4, Group 2, FGCT3.

### The impact of identity on responsibility: the importance of decision making and acting with autonomy

Legal constraints prevent students assuming responsibility for some activities but perceptions of limited competency and a sense of trepidation further limit the student role:

But I think that’s what I feel is going to be the transition is the fact that where obviously now I can’t do anything –like I can’t sort out prescriptions or anything at all like that [orientation] –I’m then going to go to the point where I can be the one that’s signing prescriptions and organising all the tests and being responsible [complication] from that side of things, whereas at the moment actually I’m not responsible for it and there’s someone above me that’s always responsible for everything that I don’t really as such have to worry about, if that makes sense [evaluation] Student ID 12.

Newly qualified doctors also emphasised how workplace limitations and the perceptions of other professionals could impact beyond the strict legal constraints on the stepwise change in responsibility:

I think nurses know that medical students can’t prescribe, they can’t really make many decisions [orientation], and so that’s where the difference is for being part of the team –‘cause the nurses don’t ask the medical students things they ask [us] now [complication]. And that’s the big difference [evaluation]…Because even today, I had a medical student on my ward and I didn’t know he was turning up at all, …but there was so much to be done, sort of admin-wise, and you give them the bloods and they’re then just waiting around and you’re sort of rushed off your feet doing other stuff [orientation] and, you know, they don’t…they don’t know how to do TTO [to take out medication], they’ve had no access to e-script…so it’s sort of difficult to engage them because you don’t know they’re coming, you don’t know where they’re at, they don’t have any of the user names or log-ins or anything[complication], so all I could really give them was bloods and cannulas to save me time [resolution]. SITE3, Group1, FGID11.

Students who managed to negotiate a more proactive role were rewarded with trust and more responsibility by junior doctors, but this often required using unofficial workarounds for the system:

…but the flip side is, I’ve had a really… med student [orientation], who was with me in gastro and now he’s come to urology and like he basically uses my log in [resolution], so he…I sent him from where I was to the…three floors down, to go and do a discharge ‘cause I knew that he would do it, and then obviously I checked it, from upstairs [evaluation], and then he printed it out and brought it to sign it, so if you can get a junior’s account details –like he reviews bloods and tells us on ward rounds… SITE 3, Group 1, FGID13.

Junior doctors are also supervised but qualification permits and requires them to develop some degree of autonomy in practice, accepting a share of responsibility for care. This is something which students anticipate but represents a change which they are unable to make without going through the process of graduation:

I know there’s people above you who should be reviewing things [orientation], but to a certain extent you are a doctor and you should be able to deal with some stuff on your own without being constantly supervised [complication]. So I do think there’s a different level of responsibility, knowing that some of the buck stops with you, rather than just saying ‘I’m only a medical student’ [evaluation]. Student ID21.

However, when the student is given responsibility it remains limited and most of the responsibility remains with the supervisor irrespective of the student’s degree of participation in patient care:

You have that responsibility as an F1 [orientation]. And you…other people, like the nurses on the ward, are looking up to you for answers sometimes[complication] –no-one looks up to students for answers –and yeah, you’re responsible for the patient’s care, you’re responsible for their condition and what happens to them so it’s mostly that, yeah, that responsibility aspect. Whereas as a final year, you’re just interested in the patient but not actually responsible for the care [evaluation]. Student ID29.

Graded exposure to responsibility is seen as beneficial though access to opportunities is limited and opportunities may be limited to tasks of low risk:

I think it’s…if you can get exposed to even a little bit of, you know, doing things yourself –like say with my diabetics’ clinic [orientation], although to everyone else you’d probably think that was dead minor ‘cause you’ve been like a doctor and things, but to me it was like a load of responsibility, checking someone’s HbA1C [complication], it makes me laugh now but when you’re in that situation, I felt that was a responsibility, a little bit, so if I can get gradual exposure, I’ll feel more comfortable [resolution]. Because it…the first patient that I saw doing that I was apprehensive…I was apprehensive about it because it was the first decision I’ve ever made really, perhaps, that [evaluation]. A minor decision, like I said. Probably out of proportion, but maybe I’m just a worrier. Student ID01.

Although students are stewarded in the completion of tasks they are prevented from taking clinical decisions and restricted in participating in the care of the sickest patients:

After qualification, when I started my first job [orientation],…things which like troubled me were during medical school we’re sort of protected and we see patients, sick patients, but we don’t take any decisions or we don’t have to take responsibility as much for those patients [complication], so we can see the sick patients and then come away while the team looks after them [complication]. But as an imminently qualified F1 or a house officer, you’re there out of hours and you’re responsible for the patient, taking decisions [resolution], so that transition was a bit difficult [evaluation]. SITE 4, Group 1, FGCT2.

Though foundation doctors are part of a larger team, there are times when they are required to make judgements about patient care without immediate availability of direct support. New doctors would give themselves permission to take responsibility in acute situations because there was nobody else to give it. In addition to the pressure of acutely changing clinical situations, nurses expected autonomous decision-making and leadership which reinforced the decision to act. This responsibility was burdensome:

But then you get this scary moment when they look at you and…they say ‘what do we do doctor?’ FYID6.

However, doctors in acute posts without the requirement to act with autonomy could perceive that their development of professional responsibility had been stunted:

And how have you all found it just starting job number two versus starting job number one?

I feel like I’ve only just started being a doctor [orientation] ‘cause I’ve been on anaesthetics and supernumerary [complication] and like I’m just essentially a student again, so I’ve only really done four days of being a doctor, it feels like [evaluation]. SITE 2, Group 2, FGID3.

Often the initial weeks post qualification were spent closing the gap between graduating students’ expectations about the role of a newly qualified doctor and the reality they discovered in post. The realisation that there was a gap between their expectations and reality stimulated negotiation of boundaries for their new role, and an increased sense of uncertainty as they tried to define their role in the messiness of busy clinical practice:

Interviewer: Is there anything else we’ve not talked about that you’d say was difficult in your transition to becoming a doctor?

FGID1– I think just maybe knowing the boundaries of what you should…what we can do as F1s maybe [orientation] –things more like speaking to families, breaking bad news as well, some people seem to be doing that when I thought we weren’t supposed to be [complication]. That kind of thing and…it’s difficult though ‘cause there’s so many scenarios, you can’t cope with them all [evaluation].

FGID2– and there wasn’t any set guidelines at all…[complication].

FGID1– it’s consultant’s preferences, isn’t it? [resolution].

SITE 2, Group 2.

### The impact of responsibility on identity

The requirement to make decisions and act with some degree of autonomy is important in accepting responsibility and defining the new identity of the doctor. However, the experience is stressful.

Interviewer: How did it feel though?

Stressful. Particularly for the first week [orientation]. Didn’t quite know what I was meant to be doing and what responsibility I was meant to have [complication]. I knew that I was registered on the GMC but I didn’t know if that meant it was okay for me to prescribe, if it was okay for me to see patients on my own, whether or not I’m meant to be thinking like a fifth year medical student or an F1 doctor [complication]. I suppose I just went for the level that I thought was appropriate, which was pretty much the same as what the nurse practitioner was doing on the ward [resolution]. And then I suppose during ward rounds, the consultant would ask me to do things, so I was getting a grasp of exactly what he wanted me to do and then when I did my other shifts on the acute medical unit, looking at the other F1s, judging what their attitudes were like and things were quite good [evaluation]. FYID3.

Step changes in responsibility come with new jobs when roles change but identity formation is an evolutionary process that is chronologically mismatched with new appointments. Furthermore, roles, responsibilities and rules of interaction within clinical teams are complex and not uniform. They vary somewhat depending on specialty, team composition and setting and are rarely made explicit:

If you have a good set of nurses that realise it’s your first day, you’re usually okay –they will then help you and they’ll tell you what normally happens [orientation].

Interviewer: You said ‘if’…?

Sometimes nurses are more like ‘well, you’re the doctor, you should know’ [orientation] and you’d be like I’ve been a doctor for a day, I don’t know [complication]. …most nurses are helpful, but you get a few who will adamantly be like no, you’re the doctor [complication], you should know and then you’re a bit stuck [resolution]. SITE 4, Group 1, DGCT4.

This resolves with accumulating experience and clarification of the responsibilities associated with the role. Taking on this new responsibility appears inextricably linked with the identity of being a doctor:

Just taking on that kind of cloak of responsibility [orientation]…it was new [complication]. I don’t think anyone can prepare you for it [evaluation]. FY ID2.

The use of the ‘cloak’ metaphor emphasises that the same person (underneath) is now able to meet their new responsibilities because of a step-change in their outward identity which comes with appointment to a junior doctor’s job. Transition involves the individual both accepting the burden and allowing change in order to reconcile themselves with their new identity and responsibility:

I think the title of doctor suddenly changes you [orientation] from somebody who doesn’t have a lot of responsibility, who might have some knowledge, who might know what they are talking about but doesn’t really have the responsibility to go with it, to suddenly being somebody who has to make a decision and bears the responsibility of that decision [complication]. That’s how I explain it to myself, this is part of that change from being just a medical student who is equipped with plenty of knowledge and some experience, to be the person who makes the decision and ultimately has to live with the consequences of that decision-making process [evaluation]. FY ID17(by telephone).

On assuming the foundation doctor role, others may perceive a new capability, whilst inwardly the same person persists. Accruing experience and success in meeting the demands, the incumbent grows into the role and gets used to carrying responsibility. Thus accepting the burden of responsibility may alter the recipient’s self-image, seeing themselves as different from the student, their near-peer, for whom they now have responsibility. The final metamorphosis may be confirmed by changes in the way team members view them, noting perceptible changes in their conduct in the professional role:

I spoke with a couple of the SHOs [orientation] and they were like you’ve changed dramatically[complication], you’re confident, you know, your timings of doing this job has improved, your mannerisms on the ward are more confident, and the consultant echoed that as well [evaluation] SITE 1, Group 2, FGID2.

On-going responsibility and the requirement to act boosts self-efficacy when decisions made are supported by the team, through action and subsequently the validation of senior colleagues. Some examples of good practice in supported responsibility were found, such as this example where the participant was encouraged to lead the team in managing an acute clinical situation:

…it made me feel good, made me feel sort of authority as a team leader and it let me know they…it made me feel reassured that somebody was there, willing to help me and if the situation did get out of hand, there’d be soon a lot more hands helping me FYID3.

To summarise, differences in supervision between a junior doctor and a student can be seen even in a relatively junior doctor’s autonomy to choose to act independently, including taking (or declining) responsibility and decision making without the absolute requirement to be directly supervised.

## Discussion

The novel contribution of this study is to illuminate the complex relationship between responsibility and identity, which occurs pre, during, and post transition to qualified practice as a doctor. We have demonstrated that it is not possible to say which comes first, identity or responsibility. Because of the complex relationship between responsibility and identity which occurs pre, during and post transition to qualified practice as a doctor the two are closely intertwined, each generating the other through successful actions in practice (Dornan 2012). Our analysis brings to the fore the meaning and implications of this for those undergoing transition in medical practice and could serve to outline how these issues could be explored in other health professions.

### Anticipating and preparing for responsibility: understanding roles, requirements and becoming part of the team

The workplace is the dominant instructional setting where learners acquire the ‘tricks of the trade’ ready for graduate practice. As observed previously (Dornan et al. 2007), learning in the workplace is variably structured and occurs through a spectrum of activity from observation to supervised participation through to autonomous practice. However, accessing the opportunities to do so is difficult. Students seek to observe and then engage in supported practice. However, roles and interactions with the clinical team vary between settings and are rarely made explicit. Expectations and the code of engagement only become apparent to students through an iterative process of trial and error during which the student establishes the level of engagement that they will be permitted within each context. Responsibility may not be simply ‘taken’ by a student but instead has to be bestowed by those holding authority and clinical responsibility for the patient. This requires students to develop an understanding of the complexities of the clinical workplace and how to engage most effectively (de Feijter et al. 2011; Mennin 2010). Before the student is permitted to engage with many clinical activities, trust and a shared understanding of what is required in terms of learning and supervision must be developed. Learning is enhanced when clinical teams provide students with ready access to learning opportunities and actively plan and promote opportunities to participate in patient care.

There is a fine line between too much and too little responsibility and perceived step-changes create ‘identity gaps’—learners need support to grow and internalise their professional identities in order to realign with new responsibilities and roles. Much of what shapes this process, for better or worse, is the attitudes and behaviours of other professionals; often small, and perhaps seemingly innocuous assumptions can have a significant impact. Our data suggests that those in supervisory roles need to be more aware and make more effort to facilitate behaviours that delegate authority to the transitioning learner whilst maintaining oversight (Walzak et al. 2019).

### The impact of identity on responsibility: the importance of decision making and acting with autonomy

The requirement to act relatively autonomously and provide direct patient care is pivotal to assuming the doctor role. This is an important step change and a qualitative difference in responsibility. Junior doctors are supervised but qualification permits and requires them to develop a degree of autonomy and to accept a share of responsibility for care: ‘the buck’ does at least now partially stop with them. Making decisions is inextricably linked with some responsibility for the consequences. This is something which students anticipate but represents a change which they are unable to make without going through the process of graduation. When students were allowed to share responsibility they frequently reported a sense of jeopardy. At times, and apparent to them in hindsight, this could be disproportionate to the size of the task. Although students are able to recognise the value in undertaking these tasks, barriers such as concerns about the legality of their authority to take on tasks were an issue. Recognising and receiving the active support of a more senior team member in overcoming these barriers was important. Both not enough and too much autonomy (feeling unsupported and alone in acute challenges due to a mismatch in needs and levels of supervision) can reduce the sense of professional development (Cruess et al. 2016) and illustrate the fine balance between support and challenge required to maximise potential learning. Commitment to attempt and master these challenges or ‘troublesome’ activities, was seen by students as helpful in developing their understanding of what it is like to ‘hold’ responsibility for patient care.

### The impact of responsibility on identity

We heard junior doctors recount their experiences of transition and the perceptible steps between accepting the mantle, the external imposition of responsibility and the requirement to act autonomously prior to the internal change in their sense of self. Successfully enacting this requirement creates identity capital (Côté 1997; Côté and Levine 2002) which accumulates and stimulates a metamorphosis when they can acknowledge their growth in self-confidence and proficiency. With this comes completion of the transition into the identity of being a doctor. The growth in confidence and the change in manner of professional interactions can be apparent to colleagues. This transformational process, applying a rehearsed but diverse skill set in a previously restricted area of practice naturally results in angst, especially given the nature of the decisions required. Once completed and particularly when acknowledged by a more senior colleague, it results in an irreversible ontological shift in the way the new doctors view themselves professionally. As such, we believe this fulfils the notion of a threshold concept (Meyer and Land 2005; Randall et al. 2018).

There is increasing evidence that transitions need to be recognised as evolutionary processes with success defined as progressive independence balanced with the fostering of appropriate team-working and shared responsibilities (i.e. co-dependence) rather than the unachievable and undesirable goal of total independence and autonomy (Yardley et al. 2018). Stepwise changes in responsibility need to be supported with time for self-perceptions of identity, including a sense of legitimacy in roles to ‘catch-up’. Increasingly these are recurrent themes in medical careers highlighted at each stage of progression from one level of training to the next and beyond.

### Strengths and limitations

Although the participants were from a number of medical schools in the UK and some were international graduates, this study was undertaken in a single locality and the educational culture and healthcare working environment may differ elsewhere.

We have analysed the intersection of responsibility and identity as perceived by students and newly qualified doctors. Our participants were not asked specifically about when things go wrong and this may explain the relative lack of stories regarding such events. This could be viewed as a strength because it avoided priming participants to focus only on parts of the process of transition but it is also possible that participants were keen not to present stories where something went wrong (stories are not neutral but inherently told with a purpose),—and participants may have wanted to emphasise their worthiness of the responsibility and identity of being a doctor. In our analysis we have sought to take a critical stance to choice and how the stories were told as mitigation for these issues but we acknowledge we have no control over the selection of stories.

### Implications for practice and research

These findings help us to understand better how new graduates might grow their professional identity and to inform models of ‘supported participation in practice’ (Dornan et al. 2007). Specifically they highlight the need to integrate greater responsibilities into undergraduate learning as they prepare to make the transition into postgraduate medicine (Cruess et al. 2014).

Our findings suggest that the clinical engagement required to prepare for transition is achieved predominantly through trial and error, an unpredictable process that may fail to ensure that students engage with the activities they will assume responsibility for upon graduation. We suggest that although the adoption of the cloak of responsibility and the new doctor identity are necessarily troublesome processes, threshold concepts, the manner in which this process occurs must be improved to avoid compromises to learning and patient safety.

Merton (1957) describes shaping “…the novice into an effective practitioner of medicine, to give him [sic] the best available knowledge and skills, and to provide him with a professional identity so that he comes to think, act, and feel like a physician”. Recently there has been a movement in postgraduate medical training towards defining ‘entrustable professional activities’ (EPAs) in training programmes and signing off a trainee at each level of supervision required (Hauer et al. 2013). This has been proposed as applicable to undergraduate medical education (Chen et al. 2015). Our results lend support for the development of more explicit scaffolding of clinical activities and the degree of clinical autonomy that may be conferred to the student. We believe that immersion in the clinical workplace coupled with the development of a framework of EPAs may help the student more effectively and safely negotiate the transition and identity formation through the graded autonomy and the sharing of responsibility. However, the complexity of the relationships revealed by this data means that this cannot be assumed and will require further research.
